# Gene expression changes in pancreatic α-cell lines following knock-out Of either CK2α or CK2α’

**DOI:** 10.1186/s40659-025-00654-x

**Published:** 2025-11-13

**Authors:** Jens Mayer, Mandy Pack, Mathias Montenarh, Claudia Götz

**Affiliations:** 1 Institute of Human Genetics, Saarland University, Bldg. 60, 66421 Homburg, Germany; 2 Medical Biochemistry and Molecular Biology, Saarland University, Bldg. 44, 66421 Homburg, Germany

**Keywords:** Protein kinase CK2, CK2α isoforms, CK2 knock-out, Gene expression profile, Pancreatic α-cells

## Abstract

**Background:**

Protein kinase CK2 is known to exist as a tetramer of two catalytic α- or α’- subunits and two non-catalytic β-subunits, or as multimers of this tetramer. Moreover, CK2α (CSNK2A1) and CK2α’ (CSNK2A2) are also active in the absence of CK2β (CSNK2B). Very little is known about specific functions of the individual subunits of protein kinase CK2.

**Results:**

In order to study the effects of CK2α and CK2α’ on gene expression, we used the *Mus musculus* pancreatic α-cell line αTC1 and two derivatives with either CK2α (KO1 cells) or CK2α’ (KO2 cells) expression knocked-out by CRISPR/Cas technology. We found numerous genes deregulated in both KO1 and KO2 cells compared to the parental cells. Applying stringent thresholds, 266 genes were found down-regulated and 153 genes up-regulated in KO1 cells, 233 genes were found down-regulated and 84 genes up-regulated in KO2 cells. Dozens of genes were found deregulated in a similar fashion in both KO1 and KO2 cells. We found altered expression of genes involved in the differentiation of pancreatic cells, including Hox genes, and in the regulation of glucagon synthesis or secretion. Moreover, many of the deregulated genes play an important role in developmental processes and in neuronal cell biology.

**Conclusion:**

Our findings reveal individual and shared functions of the CK2α and CK2α’ catalytic subunits, in particular regarding their involvement in regulating gene expression.

**Supplementary Information:**

The online version contains supplementary material available at 10.1186/s40659-025-00654-x.

## Introduction

Protein kinase CK2 plays a prominent role in the human kinome as it is essential for the regulation of various cellular processes [1]. Protein kinase CK2, formerly known as casein kinase 2, is a dual specific kinase that phosphorylates either serine/threonine or tyrosine residues in an acidic environment [2–4]. The enzyme occurs in various forms in eukaryotic cells. In addition to two catalytic CK2 subunits, CK2α and CK2α’, there is a non-catalytic CK2β subunit. Those three subunits are encoded by genes *CSNK2A1*, *CSNK2A2*, and *CSNK2B*, respectively. The three subunits can form tetramers of two catalytic and two non-catalytic subunits (CK2α_2_/CK2β_2_, CK2αα’/CK2β_2,_ CK2α’_2_/CK2β_2_), as well as oligomeric forms of the tetramer [5–7]. Remarkably, the CK2α’_2_/β_2_ holoenzyme has a reduced thermostability compared to the CK2α_2_/β_2_ holoenzyme [8]. Moreover, the affinity between CK2α’ and CK2β is significantly lower than the affinity between CK2α and CK2β [9]. In addition, CK2α and CK2α’ are active in the absence of CK2β. Different tissue specific expression, subcellular localization, enzyme activities and substrate specificities with resulting different functions have been described for all those different forms [10–13]. In addition to enzymatic properties of CK2, individual subunits bind to a large number of cellular proteins [14–17] with seemingly regulatory functions. PP2A and CKIP-1 were described to bind to CK2α but not to CK2α’ [18, 19]. On the other hand, KIF5C and the breast cancer metastasis suppressor 1 (BRMS1) bind to CK2α’ but not to CK2α [16, 17]. CK2α’ also seems to play a specific role in the regulation of the calcium channel Ca_V_1.2 at least in cardiomyocytes [20, 21]. The non-catalytic CK2β subunit, in particular, is known to have regulatory effects on other cellular proteins [22, 23].

It has long been known that CK2 kinase activity is elevated in fast-growing cells, such as tumour cells. This has resulted in an intensive search for inhibitors of the CK2-specific protein kinase activity. The large number of CK2 inhibitors, which were developed in the past, harbour more or less pronounced off-target effects [24, 25]. Inhibitors of CK2 kinase activity are not only used for treating cancer cells but also for the identification of new cellular functions of CK2 [26]. The majority of CK2 inhibitors inhibit both catalytic subunits in the vast majority of cases. Despite several attempts to elucidate individual functions of either CK2 catalytic subunit, little is known so far about their specific roles in regulating gene expression.

The CRISPR/Cas9 method provides a tool for switching off expression of individual genes in cells. We recently published a murine pancreatic α-cell line with a CRISPR/Cas9-mediated knock-out of CK2α. CK2α knock-out (KO1) cells are viable and proliferate slightly slower than the corresponding wild-type (WT) cells. Compared to WT cells, KO1 cells behave like cells treated with either CK2 specific inhibitors CX-4945 or SGC-CK2-1, and exhibit significantly reduced glucagon expression [27]. In order to obtain further insight into the individual function of CK2α and CK2α’ on the regulation of gene expression, we now generated a murine pancreatic α-cell line with a knock-out of CK2α’ (KO2 cells). These two KO cell lines together with the parental cell line enabled us to analyse the impact of CK2α and CK2α’ on gene expression compared to parental WT cells and to each other. We analysed RNA-seq data generated from the different cell lines in order to identify differentially expressed genes, and we analysed known biological functions of differentially expressed genes. We discuss selected deregulated genes in the context of known functions and published observations of CK2α isoforms. Our findings provide a deeper insight into gene regulatory functions of the two catalytic CK2α isoforms.

## Methods

### Cell culture

The murine pancreatic α-cell line αTC1 clone 6 (ATCC: CRL-2934) (WT cells) was cultivated in DMEM (1 g/l glucose) supplemented with 10% (v/v) FCS in a humidified atmosphere with 5% (v/v) CO_2_ at 37 °C. To analyse the effect of the loss of either one of the catalytic subunits of CK2 on gene expression levels in αTC1 cells, we used cells with a knock-out of CK2α (KO1 cells) [27], or CK2α´ (KO2 cells) by CRISPR/Cas9 technology. Knock-out of CK2α´ employed plasmid pD1431-Apuro:511,938 with a guide RNA for mouse CK2α´, as designed by ATUM (Newark, USA). Plasmid transfection was carried out using Lipofectamine 3000 (Fisher Scientific, Schwerte, Germany) according to the manufacturer´s instructions. After 48 h, cells were exposed to puromycin at a final concentration of 2 µg/ml, with puromycin-containing medium renewed every three days, to create a stable cell line (KO2 cells). Lack of protein expression of knocked-out CK2 subunits was verified by Western blot and functional assays.

### Western blot analysis and antibodies

For harvesting, cells were scraped off the plate in phosphate buffered saline (PBS) and sedimented by centrifugation (7 min, 4 °C, 400xg). Cells were washed with PBS and lysed with the double volume of RIPA buffer (50 mM Tris/HCl, pH 8.0, 150 mM NaCl, 0.5% (w/v) sodium desoxycholate, 1% (v/v) Triton X-100, 0.1% (w/v) sodium dodecyl sulphate) supplemented with complete protease inhibitor cocktail and PhosStop phosphatase inhibitor cocktail (Roche Diagnostics, Penzberg, Germany). After lysis, cell debris was removed by centrifugation (30 min, 4 °C, 15,000xg). Protein content was determined employing a modified Bradford method (Bio-Rad, Munich, Germany). Proteins were separated by SDS polyacrylamide gel electrophoresis in 10% or 12.5% polyacrylamide gels and blotted onto a PVDF membrane. After blocking with Tris-buffered saline (TBS) (20 mM Tris/HCl, pH 7.5, 150 mM NaCl) supplemented with 0.1% (v/v) Tween 20 (TBS-T) and 5% (w/v) BSA for 1 h at room temperature, membranes incubated with primary antibodies at a dilution of 1:1000 in TBS-T with 5% (w/v) BSA. Total Akt1 was detected with the anti-Akt1 antibody (#9272) from Cell Signaling Technology (Frankfurt am Main, Germany); Akt1 phosphorylated at serine residue 129 was detected with a recombinant anti-Akt1 (phospho S129) antibody (ab133458) from Abcam (Cambridge, UK). Mouse monoclonal antibody 1AD9 was used to detect the α- and α´- subunit of CK2 simultaneously [28]; monoclonal antibody E9 (sc-46666, Santa Cruz Biotechnology Inc., Heidelberg, Germany) detected the regulatory β-subunit of CK2. Polyclonal HNF1α antibody from rabbit (invitrogen PA5-22310, ThermoFisher Scientific, GmbH, Dreieich, Germany) was used to identify this transcription factor. Equal loading of gel lanes was verified using a rabbit polyclonal anti-GAPDH antibody (FL-335, sc-25778, Santa Cruz Biotechnology Inc., Heidelberg, Germany). Incubation with primary antibodies was done under gentle shaking overnight at 4 °C or for 1 h at room temperature. After several wash steps with TBS-T, membranes were incubated with a horseradish peroxidase (HRP)-conjugated secondary antibody for 1 h at room temperature. Detected proteins were visualized by enhanced chemiluminescence using Super-Signal West Pico PLUS Chemiluminescent Substrate (Thermo Fisher Scientific GmbH, Dreieich, Germany). “Quantity One 1-D Analysis” software (version 4.6.7) from Bio-Rad Laboratories Inc. (Feldkirchen, Germany) was used to analyse protein expression.

### Growth curves

Cells were seeded in a 24-well plate at a density of 1 × 10^5^/well. After 24 h, 48 h and 72 h, cells were detached, stained with 0.4% (w/v) trypan blue solution and counted using a LUNA™ automated Cell Counter (logos Biosystems, Villeneuve D’Ascq, France) according to the manufacturer’s protocol. Viability was recorded by the software during cell counting.

### In vitro phosphorylation

Enzymatic activity of protein kinase CK2 in cell extracts was analysed in vitro by incorporation of radioactively labelled phosphate from [γ^32^P]ATP into a synthetic substrate peptide with the amino acid sequence RRRDDDSDDD, as described in [29].

### Isolation of total RNA and RNA-sequencing

Total RNA was isolated from αTC1 WT, KO1 and KO2 cells in duplicate, using the RNeasy Mini Kit (Qiagen, Hilden, Germany) according to the manufacturer’s instructions. High-quality RNA samples with an OD 260 nm/280 nm ratio of 1.9–2.1 and an OD 260 nm/230 nm ratio of 2.0-2.2 were subjected to RNA sequencing (RNA-seq).

RNA-seq was performed by Eurofins Genomics (Constance, Germany), specifically the INVIEW Transcriptome Discover package included ribosomal RNA depletion, library preparation with mRNA fragmentation, strand-specific random-primed cDNA synthesis, adapter ligation and adapter-specific PCR amplification, and Illumina PE150 sequencing. 60 million total raw reads were generated per sample. Read statistics are summarized in supplemental Table 1.

### Identification of deregulated genes

High-quality reads (Q30, typically >95% of reads) were aligned to the *Mus musculus* genome reference sequence mm10 using STAR v 2.7.10b [30]. Alignment statistics and reads of genomic origin are summarized in supplemental Tables 2 and 3, respectively. Bam files, as provided by Eurofins Genomics, were further analysed employing Galaxy ( [31] as provided by UseGalaxy Europe (https://usegalaxy.eu).

We employed *featureCounts* ( [32] (Galaxy Version 2.0.6 + galaxy0) for assigning mapped RNA-seq reads to GENCODE release M15 (GRCm38.p5) genome annotations. Selected parameters of *featureCounts* analyses were as follows: strand information was set to unstranded, input was defined as paired-reads, fragments with only one read aligned were allowed, chimeric fragments were excluded, both split and non-split alignments were counted, Minimum mapping quality per read was set to zero. Only count Primary alignments/Ignore reads marked as duplicate were both set to “false”, the same for Allow reads to map to multiple features/Long reads/Count reads by read group/Largest overlap. Minimum bases of overlap was set to 1, Minimum fraction of read overlapping a feature/Minimum fraction of feature overlapping a read/Read 5’ extension/Read 3’ extension were set to zero each. Finally, reads were not reduced to a single position. Resulting count tables were further analysed employing *DESeq2* [33] (Galaxy Version 2.11.40.8 + galaxy0) in order to determine differentially expressed genes. Fit type for estimating size factors was set to parametric. Turn off outliers replacement/Turn off outliers filtering/Turn off independent filtering/Perform pre-filtering using beta priors were set to false each. *DESeq2* results files were employed for further analyses. Heatmaps were generated using *heatmap2* (https://rdocumentation.org/packages/gplots/versions/3.1.3/topics/heatmap.2 (Galaxy Version 3.2.0 + galaxy1), and join and cut tools, as provided, for processing of DESeq2 results tables prior to heatmap generation.

### Functional analyses of proteins encoded by deregulated genes

We made use of information provided by the Gene Ontology (GO) Consortium and PANTHER (Protein ANalysis THrough Evolutionary Relationships) Classification System (version 19) ( [34] as well as the STRING database (https://string-db.org/; version 12.0; [35]) to analyse biological processes and protein classes of proteins encoded by genes found significantly deregulated in our study. Functional information for deregulated genes was also retrieved from the Uniprot database (release 2025_02) (https://www.uniprot.org/) [36].

### Statistical analysis

Western blot analyses, growth curves and activity assays were reproduced at least three times. After testing data for normal distribution and equal variance, differences between two groups were assessed by the unpaired Student’s t-test. Results were expressed as mean ± SD. Statistical significance of *p* < 0.05 is indicated as “*”. Statistical significance of deregulated expression of genes was calculated by methods as implemented in *DESeq2*.

## Results

### Verification of CK2α and CK2α´ knock-outs

**Fig. 1:**
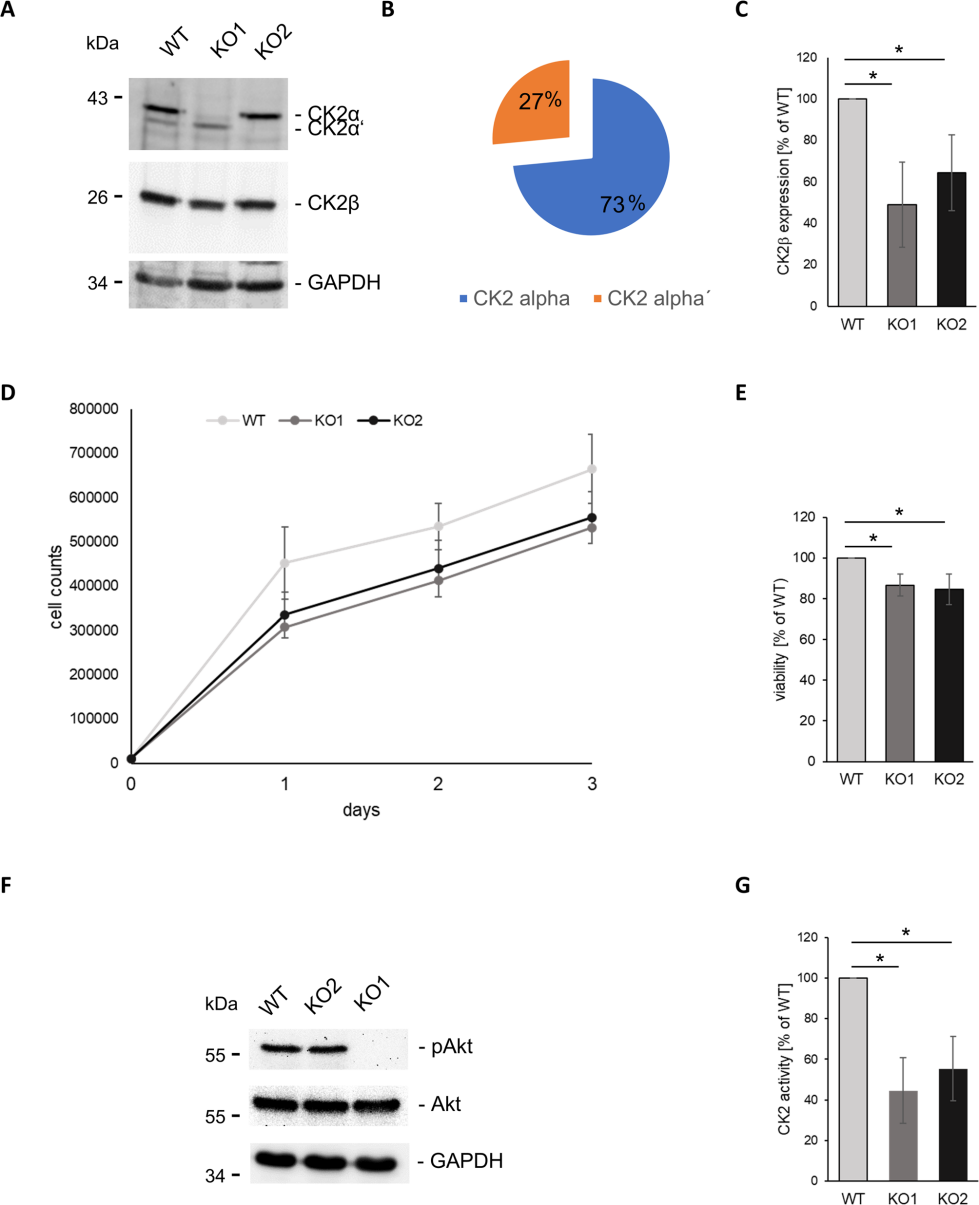
Characterization of αTC1 WT, KO1 and KO2 lines. **A** Representative Western blot of CK2α, CK2α´, CK2β, and GAPDH, expression from whole cell extracts of respective WT and KO cell lines. Mouse monoclonal antibody 1AD9 was used for simultaneous detection of CK2α and CK2α´, mouse monoclonal antibody E9 for the detection of CK2β. **B** Quantitative analysis of expression of CK2α and α´ subunits in WT cells (*n* = 3). **C** Quantitative analysis of CK2β expression in WT and KO cells after normalization to GAPDH (*n* = 3). **D** αTC1 cell were seeded in a 24-well plate at a density of 1 × 10^5^/well. The cell number was determined after 1, 2 and 3 days. **E** Cell viability of WT and KO cells, as measured by Trypan Blue exclusion assay, was assessed on day 3 after cell seeding. (**F**) Representative Western blots detecting expression of pAkt (phosphoS129), total Akt, and GAPDH from whole cell extracts of αTC1 WT, KO1 and KO2 cells. **G**. CK2 activity was determined in cell extracts using [γ^32^P]ATP and the CK2 substrate peptide RRRDDDSDDD. Activity in KO extracts is plotted relative to the activity measured in WT extracts, defined as 100%. Data are plotted as means ± SD. * = *p* < 0.05

The αTC1 cell line with a CK2α knock-out (KO1), described previously [27], was a first step towards investigating specific gene expression patterns resulting from a missing catalytic subunit of CK2 in order to track down subunit-specific gene expression and signalling pathways. We have now generated another pancreatic αTC1 cell line with a CK2α´ knock-out (KO2). We first investigated expression of all CK2 subunits in the different αTC1 cell lines (Fig. 1A). Based on amounts of detected protein the α- and the α´-subunit were efficiently knocked-out in the KO1 and the KO2 cell line, respectively. Since we used mouse monoclonal antibody 1AD9, which detects both subunits with equal affinity [28], we determined the ratio between α- and α´- subunits in the parental αTC1 cells by densitometry as 3:1 (Fig. 1B). It had been described in earlier publications that CK2 affects its own expression [37, 38]. We likewise observed that the loss of the catalytic α-subunits affected CK2β expression. CK2β was down-regulated to 50% and 65%, respectively, in KO1 and KO2 cells when compared to WT cells (Fig. 1C). We next assessed cell growth and viability of the different cell lines. Both KO cell lines proliferated slightly slower than the parental WT cell line and their viability was somewhat compromised (Fig. 1D, E).

In order to analyse CK2 kinase activity in the three different cell lines, we examined phosphorylation of Akt1 at serine residue 129 employing a phospho-specific antibody. Phosphorylation of Akt1 at Ser129 (pAkt) was no longer detectable in KO1 cells (Fig. 1F). Such phosphorylation was, however, still found in the KO2 cells. We also examined phosphorylation of the CK2-specific peptide substrate RRRDDDSDDD using cell extracts from the three different cell lines. CK2 activity was reduced to 55% in KO2 and to 45% in KO1 cells, relative to WT cells (Fig. 1G). Thus, we have demonstrated absence of corresponding catalytic subunits in the KO1 and KO2 cell lines, as well as a severe reduction of CK2 kinase activity in both cell lines.

### Deregulated gene expression in αTC1 cells with CK2α or CK2α´ knock-outs

In order to determine changes in gene expression associated with the loss of CK2α or CK2α’ catalytic isoforms, we analysed gene expression levels in αTC1 WT cells compared to gene expression levels in CK2α knock-out (KO1) and CK2α’ knock-out (KO2) cells each. High-quality total RNA was isolated from respective αTC1 wild-type and knock-out cell lines followed by RNA-sequencing (RNA-Seq) using Illumina paired-end sequencing. As per information provided by Eurofins Genomics, the 60 million total raw sequence reads per sample contained, on average, 1.58% rRNA reads, and 96.76% and 95.07% of high-quality reads and bases, respectively, and thus were of high quality (supplemental Table 1). Trimmed and filtered reads were then aligned to the *Mus musculus* mm10 reference genome sequence using STAR [30]. On average, 58.05 million reads were aligned per sample, 91.46% of reads were unique, 98.21% of reads were mapped to the reference genome, 1.8% of reads remained unmapped (supplemental Table 2). The number of reads mapping to exonic, intronic, intergenic, as well as those overlapping exons, are summarized in supplemental Table 3.

**Fig. 2:**
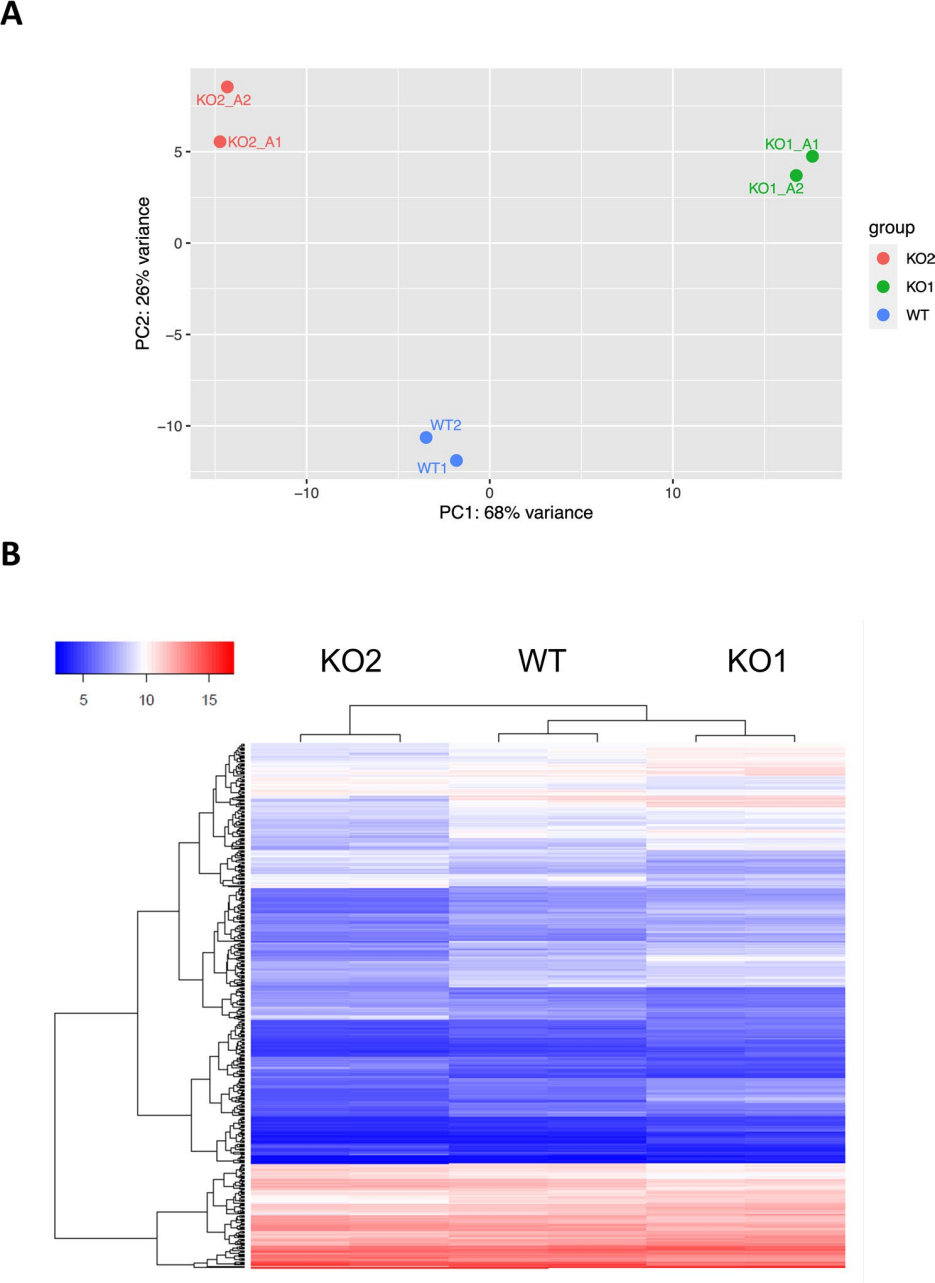
Clustering of gene expression data from replicates of WT, KO1 and KO2. **A** Result of principal component analysis (PCA). Note that replicates of WT, KO1 and KO2 display little variance each, much larger variance is observed for the group-wise comparisons. **B** Heatmap based on genes expressed in WT, KO1 or KO2, and deregulated with an adjusted p-value lower than 0.01 and an absolute log_2_-fold change of greater than 1.5. Clustering was done for columns and rows and employed the Euclidean distance method. The scale bar depicts rLog-normalized counts

We used bam files, as provided by Eurofins Genomics, for identification of genes differentially expressed between WT cells and either KO1 or KO2 cells, employing *featureCounts* and *DESeq2*, as provided by Galaxy Europe [31]. Principal component analysis demonstrated that technical replicates from each cell line showed relatively little variance among each other, yet there was considerable variance between the three cell lines each (Fig. 2A). Furthermore, a heatmap of genes which are deregulated with an adjusted p-value lower than 0.01 and an absolute log_2_-fold change (log_2_(FC)) of greater than 1.5 demonstrated that replicates from each of the three different cell lines grouped well with each other, yet both replicates were distinct from the other replicates each (Fig. 2B).

**Fig. 3:**
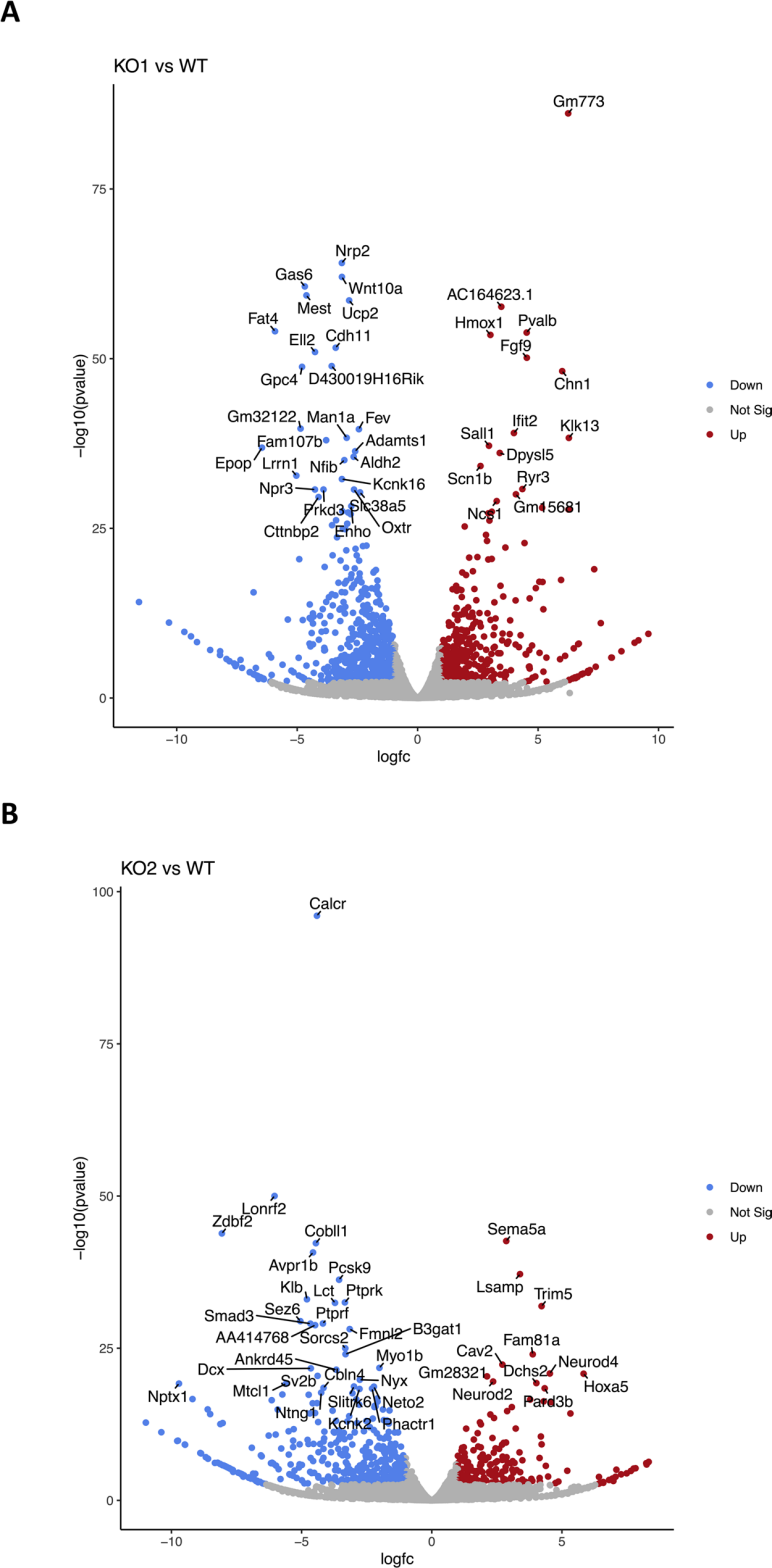
Volcano plots showing genes differentially expressed between the comparison groups. **A** KO1 vs. WT cells. **B** KO2 vs. WT cells. Genes with a significance threshold below 0.05 and a log_2_(FC) > 2 or <-2, respectively, are highlighted in red and blue. The top 40 most significantly deregulated genes are labelled each

We found numbers of deregulated genes in either KO1 or KO2 cells in comparison to WT cells (Fig. 3; supplemental Table 4). As for KO1 compared to WT, applying a threshold for log_2_(FC) of ≥ 2 or ≤-2 and an adjusted p-value of ≤ 0.01, we identified a total of 266 genes down-regulated and 153 genes upregulated in KO1 compared to WT (Fig. 3A). Notably, 23 genes became completely silenced in KO1 compared to WT cells and 14 genes became activated in KO1 compared to WT, with zero normalized counts in the respective replicates (Suppl. Table 4).

Applying above mentioned thresholds, we identified a total of 233 genes downregulated and 84 genes upregulated in KO2 compared to WT, (Fig. 3B). Similarly, 38 genes became completely silenced in KO2 compared to WT cells and 10 genes became activated in KO2 compared to WT cells, when filtering for zero normalized counts in the respective replicates (Suppl. Table 4).

**Fig. 4:**
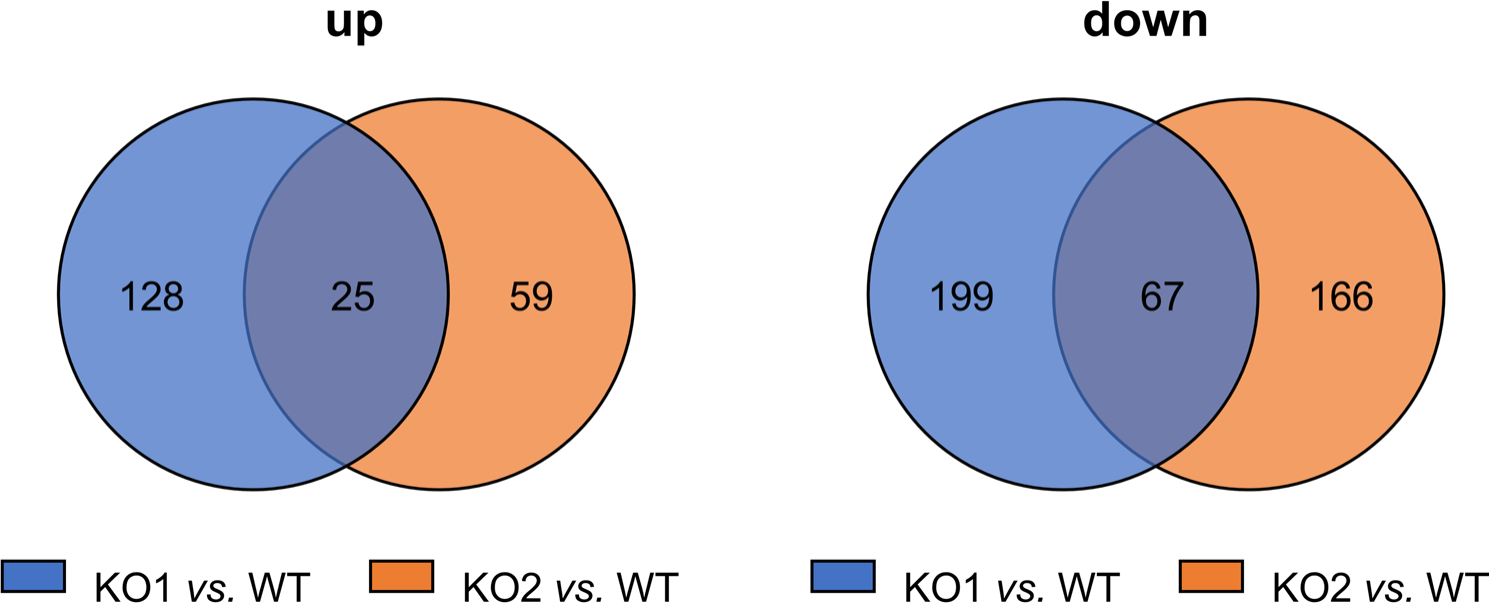
Numbers of genes regulated by the CK2α- and the α´-subunit. The Venn diagrams depict the number of genes upregulated (left) or downregulated (right) in a similar fashion in KO1 and KO2 cells compared to WT cells each

Interestingly, we found that expression of several genes was similarly affected by knock-out of either the CK2α- or the CK2α´- isoforms. Specifically, compared to wild-type each, we found 25 same genes to be up-regulated and 67 same genes to be down-regulated following knock-out of the CK2α- or the CK2α´- subunit (Fig. 4; Suppl. Table 4). This result might indicate that the CK2α- and the CK2α´- subunits can compensate each other in some cellular functions, though they are also known to have unique functions, as indicated by higher numbers of genes in non-overlapping sections of the Venn diagrams in Fig. 4 and in Suppl. Table 4 (see also below).

### Functional differences and similarities of genes deregulated in KO1 and KO2 cells

To compile information on biological functions of genes and gene products found significantly up- or downregulated in KO1 and KO2 cells compared to WT cells each, we employed STRING (Search Tool for the Retrieval of Interacting Genes/Proteins) and the PANTHER (Protein ANalysis THrough Evolutionary Relationships) classification system. Both databases analyse data in different ways and produce complementing results. We highlight below findings of potential interest in the context of CK2 biology.

**Fig. 5:**
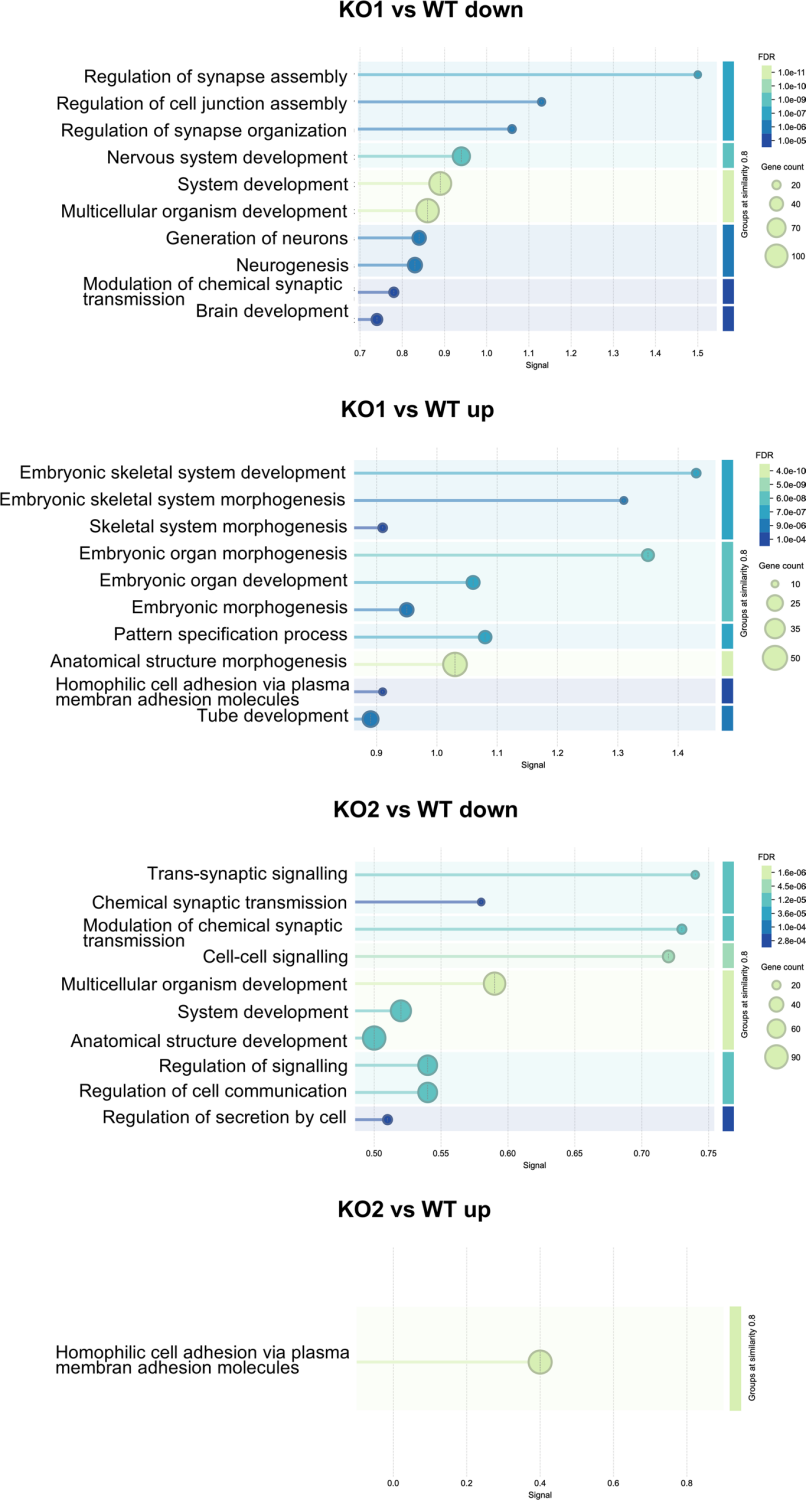
Biological Process (Gene Ontology) enrichment. Using the STRING database (https://string-db.org/; version 12.0) deregulated genes (p-adj < 0.01, log_2_(FC) >2 or < − 2, respectively) were analysed for their enrichment in biological process categories. The 10 gene ontology categories (GO-terms) with the highest signal are shown for the comparison groups KO1 vs. WT and KO2 vs. WT. Plots generated by STRING were modified in order to improve readability [35]

As for the 266 genes significantly downregulated in KO1 compared to wild-type, STRING identified significant functional enrichments for several biological processes (Fig. 5). Remarkably, processes related to development and functionality of the nervous system were among the top-ranking ones, for instance, regulation of synapse assembly (GO:0051963), regulation of cell junction assembly (GO:1901888), regulation of synapse organization (GO:0050807), nervous system development (GO:0007399), generation of neurons (GO:0048699), neurogenesis (GO:0022008), modulation of chemical synaptic transmission (GO:0050804), and brain development (GO:0007420).

As for the 153 genes significantly upregulated in KO1 compared to WT, there was no significant enrichment of neuron and nervous system-related terms seen for the genes downregulated in KO1 (Fig. 5). Instead, STRING identified, as top-most ranking, enrichments for differentiation and development of an organism, for instance, embryonic skeletal system development (GO:0048706), embryonic organ morphogenesis (GO:0048562), embryonic skeletal system morphogenesis (GO:0048704), tube development (GO:0035295), pattern specification process (GO:0007389), embryonic organ development (GO:0048568), anatomical structure morphogenesis (GO:0009653), embryonic morphogenesis (GO:0048598), skeletal system morphogenesis (GO:0048705), or homophilic cell adhesion via plasma membrane adhesion molecules (GO:0007156).

Therefore, based on our findings in αTC1 cells, CK2α appears to affect biological processes related to development and functionality of the nervous system positively, while it affects processes related to differentiation and development of an organism negatively.

As for the 233 genes significantly downregulated in KO2 compared to wild-type, generally fewer terms were found significantly enriched in STRING analyses (Fig. 5). However, we also noted an enrichment for biological processes important for the nervous system, yet, generally other processes than for KO1, e.g. trans-synaptic signalling (GO:0099537), modulation of chemical synaptic transmission (GO:0050804), and chemical synaptic transmission (GO:0007268). Moreover, genes involved in the development of an organism (GO:0007275, GO:0048731), in signal transduction (GO:0023051, GO:0010646), cell-cell-signalling (GO:0007267), and regulation of secretion (GO:1903530, GO:1903532) were found enriched.

For the genes significantly upregulated in KO2 compared to wild-type, STRING analyses did not identify significant functional enrichments within the network of those genes, apart from homophilic cell adhesion via plasma membrane adhesion molecules (GO:0007156), yet with a comparatively low signal strength of 0.4 and a false discovery rate of 0.0409 (Fig. 5).

Therefore, in αTC1 cells, CK2α’ appears to affect biological processes related to the nervous system, yet not the same processes as CK2α, and some other processes positively, while it affects a less definable set of biological processes negatively.

Of further note, STRING analyses did not find significant functional enrichments for the same 67 genes downregulated in both KO1 and KO2 cells, while the same 25 genes upregulated in both KO1 and KO2 produced relatively few functional enrichments often with relatively low strength, signal, and relatively high false discovery rate (FDR). We note, for instance, biological process homophilic cell adhesion via plasma membrane adhesion molecules (GO:0007156) and homeobox protein, antennapedia type (IPR017995). Those findings may further support the notion that CK2α and CK2α’ affect different processes in αTC1 cells, and potentially other cell types.

**Fig. 6:**
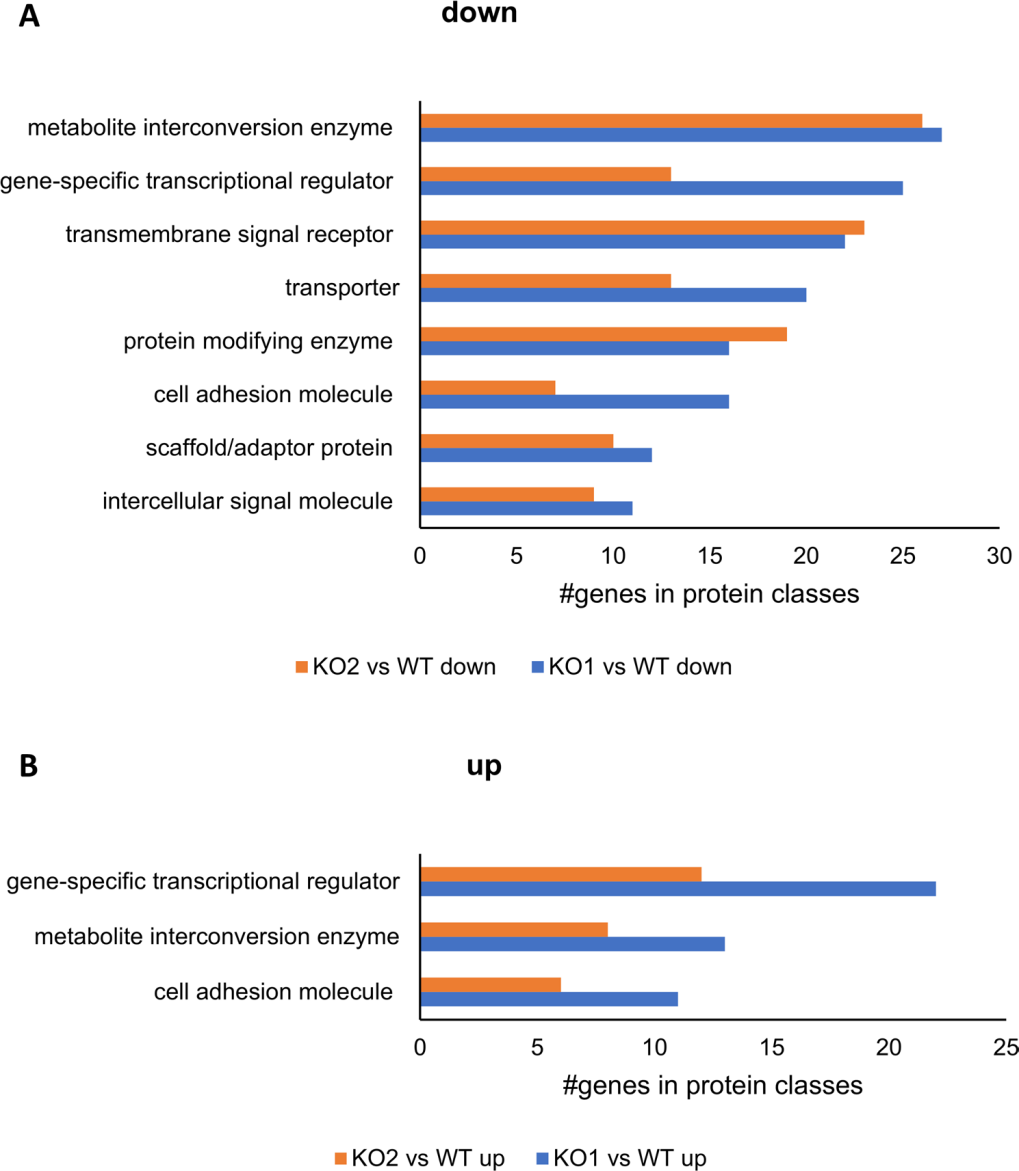
Protein classes and numbers of deregulated genes in KO1 and KO2 cells compared to WT cells each. Using the Panther classification system (https://pantherdb.org/) downregulated (**A**) or upregulated (**B**) genes from comparison groups KO1 vs. WT and KO2 vs. WT were assigned to different protein classes. (PC00069: cell adhesion molecule; PC00197: transmembrane signal receptor, PC00207, intercellular signal molecule, PC00226, scaffold/adaptor protein, PC00227: transporter, PC00260: protein modifying enzyme, PC00262: metabolite interconversion enzyme, PC00264: gene-specific transcriptional regulator)

The Panther classification system (https://pantherdb.org/) categorized genes deregulated (log_2_(FC) >2 or <-2, adj. p-value < 0.01) following knock-out of either CK2 subunit in diverse protein classes. Generally, more genes became down-regulated following knock-out of the CK2α subunit than for the CK2α´ subunit (Fig. 6A). Among the protein classes with the most downregulated genes we observed, e.g., cell adhesion molecules (PC00069), gene specific transcriptional regulators (PC00264), protein modifying enzymes (PC00260), transmembrane signal receptors (PC00197) and transporters (PC00227). Furthermore, growth and differentiation factors (intercellular signal molecule (PC00207)) like Bmp2 (log_2_(FC) = -10 in KO1 and KO2 cells) or Gdf10 (log_2_(FC) = -7,8 and − 5,4 in KO1 and KO2, respectively) were among the genes commonly downregulated by either catalytic subunit. Components of the wnt signalling machinery (intercellular signal molecule (PC00207)), specifically wnt5a and wnt10a, were downregulated following knock-out of the CK2α isoform Remarkably, CK2 has already been described to be involved in that crucial wnt-dependent developmental pathway [39, 40]. We further note metabolite conversion enzymes (PC00262) like different glycosyl-transferases as another protein class downregulated following knock-out of either catalytic subunit.

Among up-regulated genes, most members belong to protein classes cell adhesion molecules (PC00069), gene-specific transcriptional regulators (PC00264), and metabolite interconversion enzyme (PC00262) (Fig. 6B). Several homeobox transcription factors were found, such as hoxa5, hoxa6 and hoxa7 and Nkx2-5 which were up-regulated up to a log_2_(FC) of 8 in KO1 and KO2 cells, whereas homeobox proteins of the B-cluster (hoxb4, b5, b8 and b9) as well as Rhox5 became up-regulated only following the loss of the CK2α isoform.

Cell adhesion molecules were mostly represented by diverse cadherins and protocadherins. Protocadherins represent the largest group within the cadherin superfamily of cell adhesion molecules and play a critical role in the development of the nervous system [41, 42]. Remarkably, five members of protocadherin B-cluster (Pcdhb3, Pcdhb4, Pcdhb5, Pcdhb6, Pcdhb10) were found up-regulated following loss of the CK2α or CK2α´ isoform. Notably Pcdh3b was up-regulated with a log_2_(FC) of 8 and 6.9, respectively. Several other protocadherins were found upregulated following the knock-out of CK2α but not CK2α´ (Pcdh11x, Pcdhb11, Pcdhb7, Pcdhb9, Pcdhgb1).

Although we employed pancreatic α-cells, RNA-Seq analysis of knock-out cell lines identified only relatively few deregulated genes known to be directly involved in glucose homeostasis. Based on keywords in Uniprot (https://www.uniprot.org/), we identified 12 different genes to be involved in Langerhans islet function (Table 1). Some of those genes are known to be important components of a functional β-cell, others are known as key factors of α- as well as β-cell function.

**Table 1:** Genes deregulated in KO1 or KO2 cells involved in endocrine pancreas function or glucose homeostasis ^a^

gene	gene product	log_2_(FC) KO1 vs. WT	log_2_(FC) KO2 vs. WT	References
Bmp2	Bone morphogenetic protein 2	− 10.3	− 10.4	[69, 87] Growth factor of the TGF-beta superfamily, role in inflammation-induced β-cell failure, inhibits basal as well as growth factor-stimulated proliferation of primary beta cells
Ghsr	Growth hormone secretagogue receptor type 1	− 4.5	− 0.6*	[88–90] Receptor for ghrelin, expressed in β-cells, ghrelin as regulator of insulin secretion
Gpc4	Glypican 4	− 4.8	0.2*	[91] Cell surface proteoglycan, insulin-sensitizing adipokine
Hnf1a	HNF1 Homeobox A	1.3*	− 3.0	[73, 74] Transcriptional activator, maintains pancreatic α and β cell functions, controls glucagon secretion in pancreatic α-cells through modulation of SGLT1
Kcnk16	Potassium Two Pore Domain Channel Subfamily K Member 16 (TALK-1)	− 3.1	− 1.7	[81–83] In pancreatic islets, regulates frequency and duration of cytosolic Ca^2+^ oscillations coupled to secretion of pancreatic hormones; reduces δ-cell cytosolic Ca^2+^ elevations and somatostatin release, modulating signalling mechanisms that control glucagon secretion
Pak1	P21 (RAC1) Activated Kinase 1	5.1	− 1.0*	[92] role in regulation of insulin secretion in response to elevated glucose levels
Pax2	Paired box 2 protein	3.7	− 1.5*	[76] Transcription factor; linked to pancreatic development; transactivates the glucagon promoter
Pde7b	phosphodiesterase 7B	− 3.6	− 1.2	[93] influences insulin secretion
Pparg	Peroxisome proliferator-activated receptor gamma	− 2.1	− 5.7	[94, 95] Nuclear receptor, key regulator of adipocyte differentiation and glucose homeostasis, mutations associated with severe insulin resistance
Rfx6	Regulatory Factor X6 RFX6	0.3*	− 2.6	[80] Transcription factor, required to direct islet cell differentiation during endocrine pancreas development; involved in glucose-stimulated insulin secretion by promoting insulin and L-type calcium channel gene transcription; maintains gene expression and function of adult human islet α-cells
Scn3a	Sodium Voltage-Gated Channel Alpha Subunit 3	− 2.7	− 4.8	[96] required for both glucagon- and glucose-induced insulin secretion in pancreatic endocrine cells
Sstr2	Somatostatin receptor 2	− 3.1	− 4.5	[97] acts as functionally dominant somatostatin receptor in pancreatic α- and β-cells where it mediates inhibitory effect of somatostatin-14 on hormone secretion

^a^ Based on keywords and references in Uniprot (https://www.uniprot.org/)

*No significant deregulation

Although most of the genes listed in Table 1 were analysed in the context of β-cell function and effects on glucose homeostasis, several of them were also described to be involved in influencing identity and functions of α-cells, specifically Hnf1α, Kcnk16, Pax2, Rfx6, and Sstr2.

**Fig. 7:**
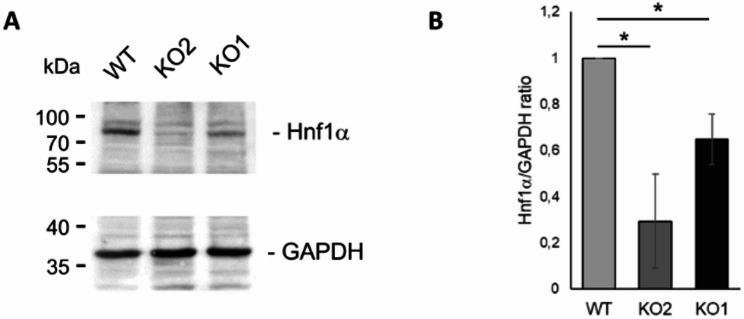
Hnf1α protein levels in αTC1 WT, KO1 and KO2 lines. **A** Whole cell extracts of respective cell lines were examined by Western blot detecting Hnf1α or GAPDH protein. **B** Quantitative analysis of expression of Hnf1α normalized to GAPDH (*n* = 3). Data are plotted as means ± SD. * = *p* < 0.05

Hnf1α is a transcription factor known as a central regulatory factor in pancreatic islet cells. We examined Hnf1α protein expression levels in αTC1, KO1 and KO2 cell lines by Western blot (Fig. 7). We found Hnf1α protein expression to be downregulated considerably in KO2 cells, much less so in KO1 cells. These results are consistent with Hnf1α gene expression being not significantly downregulated in KO1 cells, based on DESeq2 analyses, but in KO2 cells (log_2_(FC) = − 2.99; p-adj = 0.001002). Therefore, changes in Hnf1α gene expression levels were reflected by changes in protein expression levels.

## Discussion

Although protein kinase CK2 is known for approximately 70 years, little is known about the individual functions of the two catalytic subunits of this kinase. The CK2α and CK2α’ isoforms display a high degree of homology in their amino acid sequences, which made it difficult to recognize that both isoforms exert the same but also different biological activities. When knocking out CK2α in mouse, embryos exhibited structural injuries in heart and neural tube and died during embryogenesis, whereas CK2α’ knock-out mice were viable, yet male mice showed globozoospermia [39, 43]. This indicates different, important roles of the two subunits.

In the present study, we used pancreatic α-cells in which CK2α and CK2α’ were knocked out separately using CRISPR/Cas9 technology, in order to analyse the impact of either catalytic subunit on gene expression. Using a CK2-specific substrate peptide, both KO cell lines displayed a strongly reduced intracellular CK2 activity. However, the KO1 cell line demonstrated a complete loss of Akt1 S129 phosphorylation whereas, in KO2 cells this phosphorylation was still detectable. Using myoblasts, Borgo et al. observed a complete loss of Akt1 phosphorylation at serine 129 after knock-down of both catalytic CK2 isoforms [44–46]. Low amount of a weakly active CK2α’ deletion mutant was not sufficient to phosphorylate Akt1 [47]. Zonta et al. [46] analysed neuroblastoma and osteosarcoma cell lines depleted of either CK2α or CK2α’ subunit for Akt1 S129 phosphorylation and observed a different degree of reduction in different clones. Thus, Akt1 S129 phosphorylation might not only be dependent on the presence of CK2 but also on the cellular environment. Moreover, Western blot analyses with an antibody that recognized both catalytic subunits equally well demonstrated that the parental αTC1 cell line expressed three times more CK2α protein than CK2α’ protein. Therefore, loss of the CK2α´ isoform, representing only a quarter of the entire CK2 protein kinase activity, might not inevitably result in a visible loss of Akt1 S129 phosphorylation.

Protein kinase CK2 not only acts as a protein kinase, but the individual subunits interact with various cellular proteins and thus regulate functions of those proteins [10, 14, 23]. As shown here by immunoblot analysis, in addition to the respective catalytic subunit, CK2β was likewise downregulated. This indicates that the stoichiometry of the CK2 subunits is severely affected in both KO cell lines. Our findings are consistent with results having shown that CK2α knock-down in immortalized mouse neurons [44] and CK2α/α’ knock-down in myoblasts [45] reduce CK2β protein levels. In the absence of the catalytic CK2 subunits, CK2β is obviously degraded more rapidly [48]. Hence, deregulation of genes in response to the knock-out of one of the catalytic subunits has to be evaluated in the light of the simultaneous down-regulation of the β-subunit [49].

For the analysis of deregulated gene after knock-out of either CK2α isoform we set the thresholds for deregulated genes to log_2_(FC) *≥* 2 or *≤* -2, and adjusted p-value to < 0.01. We found 153 upregulated genes and 266 downregulated genes in the case of KO1, and 84 upregulated genes and 233 downregulated genes in the case of KO2. There was an overlap of the same 25 upregulated and 67 same downregulated genes between KO1 and KO2.

Analyses of known functions of deregulated genes point to different biological processes influenced by CK2α and CK2α’. Based on results from STRING analyses, we noted that CK2α affects a number of biological processes related to development and functionality of the nervous system positively, while affecting processes related to differentiation and development of an organism negatively. In contrast, CK2α’ appears to also affect biological processes related to the nervous system positively, however, other processes appeared as enriched for CK2α’ compared to CK2α. Also, in contrast to CK2α, a less definable set of biological processes was affected by CK2α’ negatively. Relatively few same down- or upregulated genes for KO1 and KO2 compared to wild-type each, combined with relatively few functional enrichments observed for those genes, provides further support for CK2α and CK2α’ isoforms influencing separate biological processes.

Deregulation of, for instance, transcriptional regulators will cause more or less directly deregulation of a whole set of downstream genes. It is reasonable to speculate that CK2α and CK2α’ may (indirectly) regulate partially overlapping or even other biological processes in other cell types with cell type-specific gene activities different from αTC1 cells. Apart from considerable downregulation of Hnf1α in KO2 cells compared to αTC1 wild-type cells (Fig. 7), a so far unknown number of genes deregulated on the transcript level may not become deregulated on the protein level, and also depending on the experimental read-out system employed for identification of deregulated genes.

As far as we know, the CK2 subunits do not regulate expression of genes by binding directly to DNA but via interactions with other cellular proteins [50, 51]. Many transcription factors are binding partners or substrates of CK2, and their transcription factor activities are modulated either by protein-protein interaction or by CK2 phosphorylation [52–55]. The changes in gene expression profiles upon downregulation of either one of the catalytic subunits are therefore rather due to indirect activities than direct interactions of CK2 with promoter regions on the DNA.

Remarkably, downregulation of CK2α in αTC1 KO1 cells led to a significant downregulation of genes involved in neuronal development and function. These findings were also observed, though in a less stringent manner, in αTC1 KO2 cells which points to the importance of CK2 for the functionality of the nervous system [56–58]. Ceglia et al. found higher levels of CK2α than CK2α’ in mammalian brain [59], whereas Guerra et al. showed preferential expression of CK2α’ in mouse brain [22]. Recently, human neurological disorders were discovered where either CK2α (Okur-Chung neurodevelopmental syndrome, OCNDS) [60] or CK2β (Poirier-Bienvenu syndrome, POBINDS) [61] is mutated, going along with low-activity or dysfunctional CK2. Both syndromes are characterized by a wide variety of symptoms resulting in developmental delay and differences in brain function which further underscores the importance of CK2 for the functionality of the nervous system.

By knocking out the catalytic CK2α- or α´-subunit in αTC1 cells, we observed upregulated expression of genes involved in cell adhesion. Most of those genes belong to the group of cadherins and protocadherins, especially the B-cluster, a group of cell adhesion proteins predominantly expressed in the brain [62]. Analysis of the role of individual CK2 subunits in an established model of immortalized neurons revealed that CK2α’ plays an essential role in increasing cell adhesion and reducing migration properties of GN11 cells. Knock-out of the CK2α subunit counteracts cell migration, inducing dramatic alterations in the cytoskeleton, which is not observed in CK2α’ KO cells [44]. These findings raise the possibility that CK2 modulates cell adhesion via transcriptional regulation of cadherins and protocadherins.

Protocadherins from the G- and D-cluster rather than the B-cluster, however, were also reported to be involved in the wnt-signalling pathway [41]. Knock-out of the CK2α isoform also downregulated wnt5a and wnt10a. Wnt5a and wnt10a serve as initiators of the canonical β-catenin dependent and non-canonical pathway, with that signalling being critical for neural development [41].

Wnt signalling plays an essential role not only in neuronal development but also in maintenance of glucose homeostasis [63, 64]. Interestingly, the serine/threonine kinase PAK1, which is upregulated by a log_2_(FC) of 5 in the absence of CK2α, mediates the cross talk between insulin and the classical wnt/beta-catenin signalling pathway and hence, regulates gut proglucagon gene expression and production of the glucagon-like-peptide (GLP-1) [65]. In addition, CK2 was reported to be directly involved in regulating the wnt pathway [66]. Thus, CK2 affects wnt signalling not only on a post-translational level but presumably also by regulating gene expression levels of important components of the wnt pathway.

When looking at critical regulators of identity and function of endocrine pancreatic cells, we identified several growth and transcription factors up- or down-regulated by the loss of CK2α isoforms.

Surprisingly, the log_2_(FC) for the expression of Bmp2 (bone morphogenic protein 2) was reduced more than tenfold in both KO lines. BMPs belong to the TGF-β superfamily and have various functions during embryonic development and adult homeostasis [67]. While they are known for their essential role in osteogenesis they are also expressed in pancreatic islets and affect proliferation of β-cells [68, 69].

Another group of genes which is strongly up-regulated in the absence of CK2α or CK2α´ are homeobox genes, which are essential for mammalian development and also for development and differentiation of the pancreas [70, 71]. Expression of Hox genes is known to vary between pancreatic α− and β− cells [71]. Hox gene products are primarily involved in the differentiation and maintenance of cell identity. The fact that these genes are upregulated upon loss of CK2 means that CK2 inhibits differentiation processes in cells.

Surprisingly, we found only a few significantly deregulated genes known to be important for the endocrine function of α-cells and the maintenance of glucose homeostasis. Our database and literature searches revealed the following genes with a possible direct impact on the functionality of α-cells, namely transcription factors Hnf1α, Pax2 and Rfx6, sodium channel Kcnk16 (Talk-1), and somatostatin receptor Sstr2.

Transcription factor hepatocyte nuclear factor 1 alpha (Hnf1α), which we found to be downregulated on the transcript level and a barely detectable protein level in the absence of the CK2α’ isoform, is expressed in pancreatic α- and β-cells [72–74] and is involved in the regulation of both, insulin and glucagon secretion [73]. Remarkably, Hnf1α induces expression of target genes Pax6 and MafB, transcription factors essential for α-cell function [75].

Together with Pax4 and Pax6, paired box protein transcription factor Pax2 belongs to a family of transcription factors that are crucial players at multiple steps of pancreatic development and differentiation. Especially Pax4 and Pax6 are known to play a leading role in regulating pancreatic islet hormones synthesis and secretion [76]. Both isoforms of Pax2 are also able to transactivate the glucagon promoter by binding to the enhancer elements G1 and G3 [77].

Rfx6 is required to direct islet cell differentiation downstream of Neurog3 during endocrine pancreas development [78] and regulates insulin secretion by modulating Ca^2+^ homeostasis in human β cells [79]. Moreover, Rfx6 is necessary for maintaining gene expression and function of adult human islet α-cells [80]. Suppression of Rfx6 impairs the exocytotic machinery in α-cells and leads to a dysregulated glucagon secretion.

In connection with a possible role in the specific function of the pancreatic α-cell lines used here, ion channels whose gene expression was affected by the loss of CK2α or CK2α’, such as Scn3a or Kcnk16, are of particular interest. Kcnk16 is a member of the potassium two pore domain channel subfamily K, also known as Talk-1. It plays an important role in the secretion of several pancreatic hormones, e.g. insulin, glucagon and somatostatin [81–83]. Kcnk16 was downregulated by a log_2_(FC) of -3.1 in KO1 cells and log_2_(FC) of -1.7 in KO2 cells. However, we note that various ion channels are either binding partners or substrates of CK2 [84]. In addition to (indirect) transcriptional regulation of the expression of ion channel-encoding genes one also has to consider direct interaction of CK2 with ion channels or an enzyme substrate interaction.

The hormone somatostatin (Sst) is produced in δ cells in the endocrine pancreas and acts as a general inhibitor of hormone secretion. It exerts its effects by binding to a G protein coupled receptor (Sstr), of which gene family members Sstr1-5 exist. In mouse α-cells Sstr2 is the prevailing isoform [85, 86]. By downregulating Sstr2 in αTC1 cells, CK2α loss may compromise the inhibitory effect of somatostatin on glucagon secretion.

Thus, CK2 influences the expression of several genes involved in sustaining the endocrine pancreatic function either by affecting the differentiation into an endocrine phenotype or by directly affecting the function of components of the machinery responsible for the maintenance of glucose homeostasis. Our findings provide novel insights into both distinct and overlapping roles of CK2α and CK2α′ in transcriptional regulation.

## Conclusions

Due to the implication of protein kinase CK2 in manifold cellular processes it was not unexpected that a larger number of genes became deregulated upon knock-out of either of the two catalytic CK2α subunits. The observed effects are most likely not only due to loss of the catalytic subunit, but also due to the accompanying reduction of the CKβ subunit. Since transcription factors and other transcriptional regulators are often targets of CK2, observed deregulated gene expression levels are very likely not always a CK2-dependent primary effect but secondary and tertiary effects eventually affecting the particular genes’ expression levels. The large number of deregulated genes participating in diverse biological processes makes assignment of a few specific biological functions of CK2α and α’ subunit difficult, on the other hand, provides further support for diverse biological processes affected by either subunit. It will be interesting to see in future investigations which, for instance, subunit-specific deregulations are primary or downstream effects, also affect protein levels in proteomic analysis. The present gene expression analyses reported here provide hints for such future research.

## Supplementary Information

Below is the link to the electronic supplementary material.

Supplementary Material 1

Supplementary Material 2

## Data Availability

RNA-seq datasets for this study were deposited at the European Nucleotide Archive (project acc. no. PRJEB94053).
