# Supplementary material for: Gene expression changes in pancreatic α-cell lines following knock-out Of either CK2α or CK2α’
Source: Biol Res. 2025 Nov 13;58:69. doi: 10.1186/s40659-025-00654-x (PMC12616906; doi:10.1186/s40659-025-00654-x)
Supplement: Supplementary file 2 — Supplementary Material 2 [file 40659_2025_654_MOESM2_ESM.docx]

**Supplemental tables 1 - 3**

**Table 1** Sequence quality metrics ^a^

| **Sample name** | **Total Raw Reads**  **[M]** | **rRNA Reads [%]** | **Total non-rRNA Reads**  **[M]** | **Total HQ Reads** | **HQ Reads [%]** | **HQ Bases (Q30) [%]** | **GC Content [%]** |
| --- | --- | --- | --- | --- | --- | --- | --- |
| KO1_A1 | 60 | 1.41 | 59.15 | 58.17 | 96.95 | 95 | 48.66 |
| KO1_A2 | 60 | 2.66 | 58.4 | 57.46 | 95.77 | 95.07 | 48.47 |
| KO2_A1 | 60 | 0.97 | 59.42 | 58.4 | 97.34 | 94.97 | 48.86 |
| KO2_A2 | 60 | 1.15 | 59.31 | 58.39 | 97.32 | 95.11 | 48.89 |
| WT1 | 60 | 2.6 | 58.44 | 57.47 | 95.78 | 95.09 | 48.42 |
| WT2 | 60 | 0.66 | 59.61 | 58.43 | 97.38 | 95.16 | 49.01 |
| mean | 60 | 1.58 | 59.06 | 58.05 | 96.76 | 95.07 | 48.72 |
| SD | 0 | 0.853 | 0.514 | 0.465 | 0.776 | 0.071 | 0.240 |

^a^ Adapted from numbers in Eurofins Expression Analysis Report. HQ: high quality, M: millions

**Table 2**: Alignment statistics ^a^

| **Sample** | **Total HQ Reads** | **Mapped Reads** | **Unmapped Reads** | **Unique Reads** |
| --- | --- | --- | --- | --- |
| KO1_A1 | 58.17 | 57.03  98.03% | 1.14  1.97% | 53.12  91.33% |
| KO1_A2 | 57.46 | 56.33  98.03% | 1.13  1.97% | 52.07  90.62% |
| KO2_A1 | 58.4 | 57.23  97.99% | 1.17  2.01% | 53.75  92.03% |
| KO2_A2 | 58.39 | 57.32  98.17% | 1.07  1.83% | 53.62  91.83% |
| WT1 | 57.47 | 56.45  98.23% | 1.02  1.77% | 51.89  90.29% |
| WT2 | 58.43 | 57.72  98.78% | 0.71014  1.22% | 54.12  92.63% |
| **mean** | 58.05 | 57.01 | 1.04 | 53.10 |
| **SD** | 0.465 | 0.534 | 0.170 | 0.923 |
| **mean %** |  | 98.21 | 1.80 | 91.46 |
| **SD %** |  | 0.003 | 0.003 | 0.009 |

^a^ Adapted from numbers in Eurofins Expression Analysis Report. Absolute numbers (millions) and corresponding percentages are given, if applicable.

**Table 3:** Reads of genomic origin ^a^

| **Sample** | **exonic** | **intronic** | **intergenic** | **overlapping exon** |
| --- | --- | --- | --- | --- |
| KO1_A1 | 45,938,231  87.11% | 5,108,393  9.69% | 1,690,284  3.21% | 3,913,632  7.42% |
|  |  |  |  |  |
| KO1_A2 | 45,572,026  88.20% | 4,450,594  8.61% | 1,648,724  3.19% | 3,811,459  7.38% |
|  |  |  |  |  |
| KO2_A1 | 46,453,013  87.08% | 5,189,210  9.73% | 1,705,275  3.20% | 3,871,504  7.26% |
|  |  |  |  |  |
| KO2_A2 | 46,661,484  87.67% | 4,849,468  9.11% | 1,713,106  3.22% | 3,888,239  7.31% |
|  |  |  |  |  |
|  |  |  |  |  |
| WT1 | 45,200,753  87.81% | 4,554,714  8.85% | 1,719,936  3.34% | 3,754,347  7.29% |
|  |  |  |  |  |
| WT2 | 46,518,250  86.62% | 5,533,036  10.30% | 1,654,536  3.08% | 3,980,451  7.41% |
|  |  |  |  |  |
| **mean** | 46,065,464 | 4,967,327 | 1,681,073 | 3,891,566 |
| **SD** | 586,079 | 409,382 | 30,377 | 79,045 |
| **mean %** | 87.42 | 9.38 | 3.21 | 7.35 |
| **SD %** | 0.006 | 0.006 | 0.001 | 0.001 |

^a^ Adapted from numbers in Eurofins Expression Analysis Report. Absolute numbers and corresponding percentages are given, if applicable.
